# Learning and Consolidation of Declarative Memory in Good and Poor Readers of English as a Second Language

**DOI:** 10.3389/fpsyg.2020.00715

**Published:** 2020-04-17

**Authors:** Kuppuraj Sengottuvel, Arpitha Vasudevamurthy, Michael T. Ullman, F. Sayako Earle

**Affiliations:** ^1^Department of Experimental Psychology, University of Oxford, Oxford, United Kingdom; ^2^All India Institute of Speech and Hearing, Mysuru, India; ^3^Department of Neuroscience, Georgetown University, Washington, DC, United States; ^4^Communication Sciences and Disorders, University of Delaware, Newark, DE, United States

**Keywords:** declarative memory, reading, second language learning, consolidation, poor readers

## Abstract

Declarative memory abilities may be important for children who are learning to read in a second language. In the present study, we investigated declarative memory in a recognition memory task in 7-to-13-year-old, Kannada native-speaking, good (*n* = 22) and poor (*n* = 22) readers of English, in Karnataka, India. Recognition memory was tested shortly (∼10 min) after encoding (day 1) and again on the next (day 2). Analyses revealed that the two groups did not differ in recognition memory performance on day 1. On day 2, the good readers improved from day 1, whereas poor readers did not. A partial correlation analysis suggests that consolidation – the change in performance in recognition memory between the 2 days – is associated with reading skills in good readers, but not in poor readers. Taken together, these results suggest that children who struggle to read in a second language may have deficits in declarative memory consolidation.

## Introduction

In many countries around the world, children receive educational instruction in a language that differs from what is spoken at home. For example, English is a common medium of instruction even in countries where in the majority of the population are not native speakers of English (Dearden, 2014). This practice may arise from a practical necessity in some cases. In India, for example, at least 22 languages are officially recognized by the national government as of 2008. In order to have a unified education system in the face of such linguistic diversity, children in India are taught English and Hindi along with another modern language of the country.

Indeed, English is considered to be an important language for academic advancement in India. In order to ensure that students meet the standards for English proficiency by the time they reach university, English is used as the primary medium of instruction in the majority of schools. However, less than 1% of the Indian population report English as their native language (2011 census, Chandramouli, 2011). Thus, academic success in India largely depends on children’s successful acquisition of spoken English as a second language as well as reading in English as a second language. However, there is little to no investigation in the extant literature of the children who struggle to attain literacy under such conditions, either in India or elsewhere.

The present study therefore provides a preliminary examination of Indian children who struggle to learn how to read in English. Specifically, we are interested in the declarative learning and consolidation abilities of these children relative to children who are good readers of English as a second language. This focus is motivated by selected insights from the literatures on reading disorder and of reading in a second language (L2), as summarized just below.

### Reading Disorders in Readers of English as a First Language

In children who learn to read in their native language, reading disorder (RD) is often characterized as significant difficulties in the age-appropriate attainment of reading that are not attributable to differences in educational opportunities, non-verbal intelligence, or an identifiable disease or disorder that might otherwise account for the reading problems. A common criterion for RD is performance of less than 1.5 SD below the mean on a standardized test of reading, which results in about 5–10% of the population being identified as disordered (Shaywitz et al., 1990).

A widely accepted view regarding the etiology of RD is that underlying problems in phonology lead to difficulties with written word recognition and phonological decoding, that is, with using letter-sound mapping knowledge to decode novel words (Bishop and Snowling, 2004). Additionally, RD is associated with deficits in areas such as working memory (Smith-Spark and Fisk, 2007), executive function (Brosnan et al., 2002), motor function (Nicolson et al., 2001), and impairments of sequence learning (see Ullman et al., 2020, for review).

Recent theories posit that at least some of these problems in children with RD may be related to a general deficit in learning ability, in particular of procedural memory and its underlying neural substrates (Nicolson and Fawcett, 2007; Ullman et al., 2020). Indeed, children with RD in their first language (L1) demonstrate weaknesses in procedural memory (Nicolson and Fawcett, 2007; Ullman et al., 2020). It has been argued that procedural memory system abnormalities can lead to the reading problems found in RD, for example, by affecting grapheme-phoneme conversion and speech-sound representations (Nicolson and Fawcett, 2007; Ullman et al., 2020).

Within this framework, declarative memory is also posited to play a role. This system underlies the learning and memory of a wide range of information, including information about episodes as well as general world knowledge. Evidence suggests that it also underlies vocabulary learning (Hamrick et al., 2018), which is important for reading (Lervåg and Aukrust, 2010; Li and Kirby, 2015; Şen and Kuleli, 2015). Declarative memory is proposed to be intact – and possibly enhanced – in children with RD (Ullman et al., 2020). It is argued to play a role not only in typical reading, but also a compensatory role for the posited deficits in procedural memory and reading (Hedenius et al., 2013a, Ullman and Pullman, 2015).

Though there is some empirical support for the idea that declarative memory encoding is a relative strength in children with RD (for a recent review, see Ullman and Pullman, 2015), there are emerging reports that offline consolidation of declarative memory may be negatively affected. Offline consolidation is a process by which initially weak memories become strengthened and/or resistant to interference (see Diekelmann and Born, 2010, for review); this is thought be an important process that, for declarative memory, appears to occur optimally during sleep (Marshall and Born, 2007). The benefits of sleep for declarative learning that are observed in typical readers are not as apparent in children with RD (Smith et al., 2018). Moreover, children with RD have been documented with atypical sleep architecture (Mercier et al., 1993; Bruni et al., 2009; Carotenuto et al., 2016). In other words, even if the initial encoding of declarative memories is spared, there remains the possibility that children with RD may experience difficulty in the subsequent consolidation, and thus retention, of those memories over time.

### Readers of English as a Second Language

An emerging literature has examined how the development of reading varies between reading in L1 and L2 (Koda, 2007). For instance, reading in L2 appears to rely less on decoding (conversion of print into sound) than in L1, and more on a combination of semantic knowledge that has been acquired under both L1 and L2 (Tan et al., 2003). Similarly, it has been observed that L2 reading proficiency relies on L2 language knowledge, such as vocabulary (Pulido and Hambrick, 2008; Verhoeven, 2010; Verhoeven et al., 2017). This view is supported by neuroimaging evidence that L2 readers with higher reading proficiency recruit their lexico-semantic network more during L2 reading tasks, as compared to those with poor L2 reading proficiency (see Li and Clariana, 2019; Verhoeven et al., 2019, for reviews). Thus, while vocabulary is important for reading in both L1 and L2 (Tunmer and Chapman, 2012), reading ability in L2 may vary according to the factors that influence L2 vocabulary acquisition (Verhoeven, 2010; Verhoeven et al., 2017).

Declarative memory may be one such factor. This idea is motivated by findings that suggest that both vocabulary and grammar in L2 may have a tendency to be learned in declarative memory, particularly during early phases of learning (Hamrick et al., 2018). Therefore, it seems that the ability to learn and consolidate information in declarative memory may be important during the early learning of L2, and by extension, during early phases of learning to read in L2, particularly when the L2 is not introduced until primary school. Furthermore, under the framework that deficits in reading might stem from problems in general learning and memory (Nicolson and Fawcett, 2007; Ullman et al., 2020), we might suspect that problems with reading in L2 may stem from more general deficits in declarative memory.

### The Current Study

While we hypothesize that children who struggle with learning or retention in declarative memory are likely to experience difficulty learning to read in L2, there has been little empirical exploration of readers who struggle to read in a second language. Therefore, the current study was designed to investigate the relationship between declarative memory learning and consolidation on the one hand, and reading on the other, in a group of children who are learning to read in English as a second language. Specifically, we focused on a group of Kannada-speaking children for our investigation. Kannada is a Dravidian language spoken in Karnataka, a coastal state in southwestern India. Kannada is spoken at home and at most workplaces by approximately 44 million native speakers (2011 census, Chandramouli, 2011). The main medium of academic instruction in Karnataka is in English; however, the population tends to speak Kannada outside of the school environment. In other words, while individuals in this region are exposed to English text from an early school age, oral language proficiency tends to remain Kannada-dominant.

In the present study, we probed declarative memory-based learning and retention (as used in Hedenius et al., 2013b) in a group of Kannada-speaking children with good reading (GR) or poor reading (PR) performance of English as a second language. Specifically, we assessed their declarative memory via performance on a non-verbal recognition memory task shortly after learning (session 1), and again to assess retention on the next day (session 2). Given the relationship between second language learning and declarative memory (Hamrick et al., 2018; Ullman, 2020), we hypothesized that declarative learning ability may be compromised in PR. We were also interested in potential group differences in changes to task performance over the two sessions, given that sleep and offline declarative memory consolidation has been found to be atypical in individuals with disordered reading in their native language (Mercier et al., 1993; Bruni et al., 2009; Carotenuto et al., 2016; Smith et al., 2018). Finally, we hypothesized declarative memory to be predictive of reading ability, given the importance of declarative memory for lexical knowledge, and the importance of lexical knowledge to reading in L2.

## Materials and Methods

### Participants

A total of 44 participants were recruited from Mysuru district in the Karnataka state of India. All participants spoke Kannada as their first language, and were exposed to English from an early age (around 5 years old) as the language of instruction. Participants were tested at the All India Institute of Speech and Hearing (AIISH), and the procedure complied with the ethical guidelines for bio-behavioral research at AIISH. The WHO10 disability questionnaire (Singhi et al., 2007) was administered in order to ensure that participants had no sensory, motor, or notable developmental deficits (Attention Deficit/Hyperactivity Disorder, Autism). In addition, no participant had a reported history of significant language delay in L1 development. In other words, observed reading deficits within this sample are not obviously attributable to a language disorder, at least as measured in the L1 (to note, reading problems may yet be linked to weaknesses in English proficiency; Alderson, 1984). All of the participants obtained a standard score of 85 or above on Raven’s Colored Progressive Matrices (Raven and Court, 1986), indicating that their non-verbal abilities were in the typical range.

Children were classified into groups of “good” and “poor” readers. We are not aware of any standardized tool for assessing reading abilities in Kannada-speaking children whose medium of instruction at school is English. Therefore, to be classified as a “poor” reader, the child had to meet both of the following criteria. First, children in the poor reader cohort were recruited from those who were referred to the AIISH for services, by parents or teachers who reported the child as struggling in school with reading and writing in English. In addition, these participants had to obtain scores of lower than 1.5 SD below the mean for 9-year-olds on the word reading and spelling subsection of the Dyslexia Assessment Profile for Indian Children (DAPIC, Kuppuraj and Shanbal, 2012). The DAPIC is an instrument that assesses various literacy-component skills (e.g., phonological awareness, handwriting) in English. The spelling subtest contains 40 target items that include both real words and pseudowords that follow regular phonotactic rules of English. The items are auditorily presented once in isolation, again in a sentence, then again in isolation, and the children are instructed to write them down. For the reading subtest, children are asked to read aloud from a list of 70 regular and irregular words. For both subtests, items were scored by whole words as correct or incorrect. Please contact the Director, AIISH (Professor M. Pushpavathi, director@aiishmysore.in) for access to a copy of the test and stimulus materials.

Children who did not meet both of these criteria were classified as good readers. Reading abilities in L1 were not tested, due to a lack of standardized measures to assess reading in Kannada script. We note that over 80% of the curriculum uses English as the medium of instruction, and reading abilities in English have a greater impact on academic achievement than reading abilities in L1 in this context. Moreover, literacy milestones in Kannada script often lag behind those for English. Thus, while an unstandardized measure of reading in L1 may have been helpful, we reasoned that, at least for a subset of our sample, a measure of reading ability in L1 may reflect, rather than inform, ability to read in L2. See Table 1 for a summary of participants and pre-testing details.

**TABLE 1:** Group characteristics and reading performance.

	PR (*n* = 22, 5 Females)			GR (*n* = 22, 11 Females)			Comparison	
Variable	Mean	SD	Range	Mean	SD	Range	*t* (42)	*p*
Age (years)	11.63	1.89	7–16	12.33	1.26	10–14	1.22(36.74)	0.157
Non-verbal IQ	101.05	10.66	88–125	107.50	6.12	100–119	2.48(33.47)	0.019*
Word Reading (max.70)	13.77	6.10	3–29	55.91	3.70	50–61	27.69(34.61)	< 0.001***
Spelling (max. 40)	8.95	4.25	2–19	27.77	2.44	21–33	18(33.56)	< 0.001***

Performance on the reading and cognitive assessments, by group. Non-verbal IQ was assessed using Raven’s Colored Progressive Matrices (Raven and Court, 1986), and is expressed in standard scores. Reading assessments were the word reading and spelling subtests of the Dyslexia Assessment Profile in Indian Children (Kuppuraj and Shanbal, 2012). Scores are expressed in raw numbers correct. PR, Poor readers in English; GR, Good readers in English. *denotes statistical significance at 0.05 level, and *** at 0.001 level.

### Procedure

A non-verbal recognition memory task adapted from a task developed by Ullman and colleagues (see Hedenius et al., 2013b; Lukács et al., 2017) was used to assess learning and retention in declarative memory. The task was implemented here with PsychoPy (Peirce, 2008), Builder version 1.83.00. The experiment was administered on a laptop PC computer with a screen size of 15.6 inches.

The stimuli from the original task were adapted to the participants in this study by removing items that were likely to be unfamiliar to children in the target population being examined (see Kuppuraj et al., 2016 for a description of the same task). Thus, the original set of 128 items was reduced to 120 items. These 120 items were black-and-white line drawings of real and made-up images (60 real and 60 made-up). These were divided into three object sets: one for the encoding phase and one for each of the two subsequent recognition phases, with equal numbers of real and made-up items in each set.

During the incidental encoding phase, participants were told that they would see a series of objects, and were instructed to indicate if the object is “real” (press “1”) or “made-up” (press “0”). In each trial, a crosshair (+) appeared in the center of the screen for 250 ms, followed by an object image. Each object image was presented for 2000 ms regardless of when the participant indicated a response, to ensure all objects were presented for the same duration. Participants completed five practice trials, followed by 60 experimental trials (30 real and 30 made-up).

Participants completed two phases of recognition. One was completed 10 min after the encoding phase, and the second phase was completed 24–48 h after encoding. In each recognition phase, participants were told that they would again see a series of images, and were instructed to indicate if they had seen the object before during encoding. Different subsets of 30 familiar (15 real and 15 made-up) items were used as target items across the two recognition phases (together with different sets of 30 foils), in order to ensure that changes in performance between the two recognition tests are not attributable to additional encoding of images during the first recognition phase. The stimulus sets used for encoding and recognition phases were counterbalanced. Prompts remained on the screen for the duration of the experiment to remind participants of the key-response correspondences.

### Analyses and Results

Analyses were performed using R version 3.3.2. For our index of recognition accuracy, the percentage of correct trials for the “seen” (target) and “unseen” (foil) items were transformed to *d’* [z(hit) – z(false alarm)] for “real” and “made-up” items separately; see Macmillan and Creelman (2004). A *d’* of zero indicates chance performance and a *d’* of four indicates near-perfect performance. In order to avoid infinite values during the data transformation, a *d’* score of 4.5 was set as the ceiling. Please see Supplementary Material for a copy of the data.

#### Encoding Performance

In order to determine if performance differed between the two groups during encoding, independent samples *t*-tests were performed on the *d’* scores obtained during the encoding phase. The groups did not differ significantly on either *d’* scores (PR: *M* = 3.03, *SD* = 0.67; GR: *M* = 3.17, *SD* = 0.46); *t*(1, 37) = −0.82, *p* = 0.42), suggesting that the groups performed comparably during incidental encoding.

#### Recognition Performance

**FIGURE 1:**
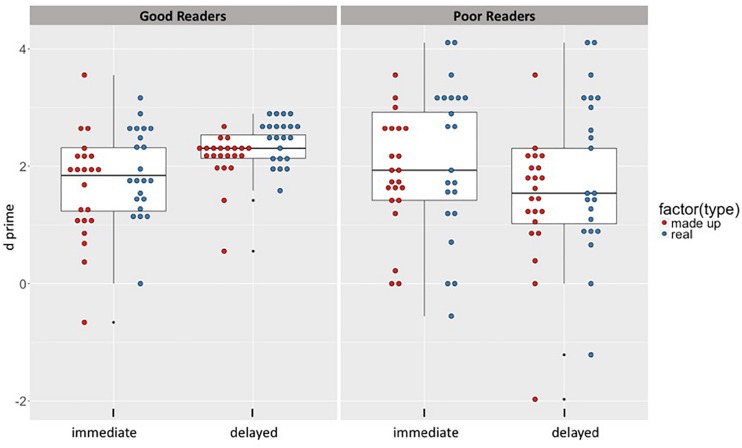
Recognition performance by Group, Session, and Object type. On average, the good readers appear to make performance gains on recognition performance across days. In contrast, the poor readers appear to decline in performance.

An important objective of this study was to determine if recognition performance changed across days, and if this pattern of potential change differed between good and poor readers. Please see descriptive summary of experimental task performance in Table 2. To address this question, a 2 × 2 × 2 (group by session by object type) analysis of variance (ANOVA) was performed on the recognition *d’* scores, with age and IQ as covariates. This revealed one main effect, of object type (*F*(1,42) = 9.97, *p* < 0.001, *η^2^* = 0.03) with better performance on real items than on made-up items over both recognition phases (real: *M* = 2.1, *SD* = 1.05; made-up: *M* = 1.76, *SD* = 2.1), with a small effect size. Additionally, one significant interaction was obtained: a group by session interaction (*F*(1,42) = 9.13, *p* < 0.001, *η^2^* = 0.05), with a small effect size. In order to determine the source of the interaction, paired comparisons were conducted on the *d’* scores across sessions for each group separately (Holms-Bonferroni correction applied). These comparisons revealed that while the GR group demonstrated a significant performance gain between day 1 and day 2 [mean difference delayed-immediate = 0.51, 95% CI (0.24, 0.79), *p* < 0.001] (see Figure 1), the PR group did not [mean difference delayed – immediate = −0.33 (negative value indicating reduction), 95% CI (−0.72, 0.05), *p* = 0.09]. No other main effects or interactions were significant (see Table 3).

**TABLE 2:** Descriptive summary of task performance by group.

Good Readers			Poor Readers				

Session	Type	*n* = 22	*n* = 22	*t*	*df*	*p*	*Cohen’s d*
Day 1	*real*	23.64(2.94)	24.05(4.90)	0.50	42	0.618	0.15
	*made up*	22.77(3.64)	23.36(4.12)	0.34	42	0.739	0.10
	*average*	23.21(2.72)	23.71(4.20)	0.47	42	0.642	0.14
Day 2	*real*	25.27(1.35)	23.05(5.10)	–1.98	42	0.054	–0.60
	*made up*	23.27(2.05)	20.45(4.13)	–2.84	42	0.007*	–0.86
	*average*	24.28(1.24)	21.75(4.14)	–2.71	42	0.01*	–0.82

Means and standard deviations of recognition memory performance on day 1 and day 2, expressed in *d*’. * indicates statistical significance at.05 level following Holms-Bonferroni correction for multiple comparisons.

**TABLE 3:** Group by Session by Object type ANOVA on accuracy performance.

Accuracy (d’)						
				Covariates: IQ, Age, encoding efficiency		

Effect	*F*	*P*	*η^2^*	*F*	*p*	*η^2^*
Group	0.899	0.35	0.011	0.893	0.350	0.012
Object Type	9.966	0.003	0.031	1.564	0.218	0.002
Session	0.408	0.526	0.002	0.408	0.526	0.002
Group by Object Type	0.204	0.654	0.001	2.817	0.101	0.004
Group by Session	9.135	0.004	0.049	9.135	0.004	0.053
Object Type by Session	0.588	0.447	0.001	0.588	0.447	0.002
Group by Object type by Session	0.269	0.607	0.001	0.269	0.607	0.001

*2 × 2 × 2 mixed ANOVAs conducted on performance accuracy. In recognition accuracy, there is a significant Group by Session interaction. This interaction appears to be driven by performance gains in good readers (GR) that is absent in poor readers (PR).*

We followed up this analysis with two additional independent samples *t*-tests (2-tailed), comparing the average recognition performance across groups on day 1 and day 2. After Bonferroni correction, we found that recognition performance was higher in GR than PR for made-up items, and also on average, on day 2. There were no differences across groups on performance on day 1. See summary of experimental task performance in Table 2.

#### Partial Correlations

In order to determine the relationship between reading measures and change across days in recognition memory accuracy, partial correlations were conducted between a) performance change across the 2 days in *d’* and b) reading abilities (accuracy at word reading and spelling, based on raw values), with age and IQ and encoding performance as covariates, for each group separately. The GR group, after Holms-Bonferroni correction for multiple comparisons, showed a performance change that was not associated with word reading (*r* (22) = 0.44, *p* = 0.060), but significantly associated with spelling (*r* (22) = 0.56, *p* = 0.013). Changes in recognition accuracy were uncorrelated with literacy ability in the PR group (word reading: *r* (22) = −0.10, *p* = 0.676; spelling: *r* (22) = 0.02, *p* = 0.928).

In sum, although the GR and PR groups performed comparably during the encoding phase, they differed in recognition memory, specifically on Day 2. In recognition accuracy, a group by session interaction was observed, driven by significant gains in accuracy between day 1 and day 2 in good readers, with no changes in performance in poor readers. Moreover, declarative memory consolidation appears to be associated with literacy skills in good readers, but not in poor readers.

## Discussion

In the present study we examined the learning and offline consolidation of non-verbal declarative memory in an understudied sample of good readers (GR) and poor readers (PR) of English, where English was their second language. The findings indicate that the PR of English as a second language were similar to that of GR in their immediate encoding of information in declarative memory, but may be compromised in their ability to consolidate that information. The present study also demonstrates a positive relationship between reading and the consolidation of information learned in declarative memory in the GR group, but not a significant relationship between these skills in the PR group. This suggests that declarative memory may be an important memory system for reading in a second language, at least for good readers. The lack of this association in the PR cohort may suggest that these children may be using a different strategy from the GR cohort. This possibility may highlight a potential intervention point, and warrants further investigation.

It is important, however, to note that the variability in declarative memory was much larger in the PR cohort on both days. In other words, despite a lack of group-level gains in declarative memory performance across days, our PR sample may have included individuals who improved in performance over time. This may point to heterogeneity in our PR sample, a subset of whom that may have a different etiology underlying their deficit.

It is unclear how this population of PR relates to children with RD in their native language. Given the relationship between L2 spoken language skills and L2 reading skills (Verhoeven, 2010; Verhoeven et al., 2017), and between declarative memory and L2 (Hamrick et al., 2018; Ullman, 2020), we might have suspected a declarative memory deficit in the PR cohort immediately after learning. Instead, we observed that declarative memory may be intact on the first day, consistent with reports that children with RD may have relatively strong – maybe even enhanced – declarative learning abilities (Hedenius et al., 2013b). Moreover, the distinct lack of consolidation effects in the PR cohort is consistent with reports that children with RD have atypical sleep, and subsequent consolidation effects (Mercier et al., 1993; Carotenuto et al., 2016; Smith et al., 2018). Given these parallels in observations between RD and the PR group examined here, direct tests of RD models in such poor readers of a second language may be warranted in the future.

In addition, the present work may have implications for learning to read in a second language in general. Despite the growing literature that suggests that the etiology of RD is fundamentally a learning and memory deficit (Nicolson and Fawcett, 2007; Ullman et al., 2020), we are only in the early stages of learning the specific contributions of domain-general learning and memory systems to the development of literacy. In a recent, longitudinal investigation, it was observed that learning abilities in declarative memory predicted reading ability in English in first grade, while learning abilities in procedural memory predicted reading ability in second grade (Earle et al., 2020). Neither type of learning ability predicted reading performance beyond the first 2 years of formal education, which was interpreted as evidence of other (e.g., linguistic) processes taking over the development of literacy beyond those initial stages of learning. The current findings indicating that declarative memory predicts reading ability in typical readers who are comparatively older (mean age 12.33), may suggest that declarative memory remain important for learning to read in L2 for a longer duration of time. This interpretation resonates with the findings in spoken language development (Hamrick et al., 2018; Ullman, 2020) that suggests that declarative memory may be critical for the learning of more aspects of language in L2 than in L1. This also raises interesting questions about the potential for differences across monolinguals and bilinguals in consolidation-related sleep architecture (see Tamminen et al., 2013, for evidence that the characteristics of the learning occurs during wake state alters spindle density in the subsequent period of sleep).

There are several important limitations to acknowledge with the present study. First, this data set does not include information about language proficiency, either in L1 or L2. Such information could help elucidate potential parallels between PR and RD subtypes, such as dyslexia (problems in decoding) and specific reading comprehension disorder. We also did not gather information regarding differences in the quality and quantity of English language exposure, which may play a role in predicting L2 reading proficiency. Moreover, we only conducted a limited number of reading assessments in order to confirm the status of the children referred for reading intervention as poor readers. Thus, the present dataset does not provide a detailed profile of reading ability. Had we done so, we may have observed various subtypes of disordered reading in our PR cohort. Such existence of subtypes could aid in the interpretation of the wider variability in declarative memory in PR than GR (see above). Thus, future investigations into this population should include measures of a broader set of reading abilities, in Kannada as well as in English. Future studies should enrich this initial examination ofKannada-speaking, struggling English readers with more detailed characterizations.

An additional important limitation is we lack that the times of day and number of hours between sessions, which were not controlled across individuals. We also did not assess the quality or duration of the sleep that took place between sessions, or of habitual sleep quality. Therefore, this dataset does not determine if the performance decline in the PR group is attributable to differences in retention/memory consolidation over wake or sleep states. Time of learning and retest will need to be controlled in future studies in order to account for potential diurnal effects contributing to learning and retention. By isolating an offline period of sleep as the critical factor for consolidation, we may begin examining the role of sleep quality as a contributing factor to differences in memory consolidation across good and poor readers.

In sum, we investigated declarative memory and its relation to literacy in school-age children in Karnataka. Although their dominant oral language is Kannada, their primary medium of instruction at school is English, which is required to achieve academic success. Thus, the present data provides an initial window into factors underlying literacy failure in children who learn to read primarily in a language other than their native language.

## Data Availability Statement

The data for this study is included in the Supplementary Material.

## Ethics Statement

The studies involving human participants were reviewed and approved by the All India Institute of Speech and Hearing Ethics Committee. Written informed consent to participate in this study was provided by the participant’s legal guardian/next of kin for the poor reader group, and by the school headmaster and/or teachers of participants in the good readers group.

## Author Contributions

This work was carried out at the All India Institute of Speech and Hearing. KS designed the study. KS and AV implemented the study under the direction of KS. KS and FE analyzed the data. KS, AV, MU, and FE prepared the manuscript. AV, MU, and FE finalized the manuscript following the death of KS. We feel the loss of KS keenly, and we are honored to see his research efforts through to dissemination.

## Conflict of Interest

The authors declare that the research was conducted in the absence of any commercial or financial relationships that could be construed as a potential conflict of interest.
